# A case report on lipofuscin deposition in a graft biopsy two years after kidney transplantation: an insignificant bystander or a pathogenic benefactor?

**DOI:** 10.1186/s12882-019-1569-6

**Published:** 2019-10-17

**Authors:** Vivian W. Y. Leung, Sarah-Jeanne Pilon, Pierre O. Fiset, Shaifali Sandal

**Affiliations:** 1 Faculty of Medicine, McGill University, 1001 boul Decarie, Montreal, Quebec H4A 3J1 Canada; 2 Department of Pathology, McGill University Health Centre, 1001 boul Decarie, Montreal, Quebec H4A 3J1 Canada; 3 Division of Nephrology, Department of Medicine, McGill University Health Centre, 1001 boul Decarie, Montreal, Quebec H4A 3J1 Canada; 4 Research Institute of the McGill University Health Centre, 1001 boul Decariel, Montrea, Quebec H4A 3J1 Canada; 5 Royal Victoria Hospital Glen Site, D05-7176, 1001 boul Decarie, Montreal, QC H4A 3J1 Canada

**Keywords:** Kidney transplantation, Lipofuscin deposition, Graft biopsy, Amiodarone, Rejection, MICA

## Abstract

**Background:**

Lipofuscin deposition is a characteristic manifestation of aging. There is very limited literature in humans and in animals describing these deposits in native kidneys. Overall, it is thought to be non-pathogenic and successful transplants from a donor with lipofuscin deposits have been reported. We present the case of a patient who underwent a kidney transplant and a for-cause biopsy post-transplantation incidentally revealed lipofuscin deposition.

**Case presentation:**

A 48-year old gentleman with a past medical history of diabetes, hypertension, coronary artery disease, and ischemic and then hemorrhagic cardiovascular accident underwent a successful kidney transplant. His donor was an expanded criteria donor with no major past medical history. Post-transplant course was complicated by delayed graft function requiring one dialysis treatment for hyperkalemia. After that he had an uneventful course and achieved a baseline creatinine of 1.2 mg/dL, with no proteinuria. On a routine 19-month follow-up he was noted to have proteinuria and an antibody against the major-histocompatibility-complex class I-related chain A. A graft biopsy revealed acute antibody-mediated rejection and impressive lipofuscin deposition. He was subsequently treated with an antibody-mediated rejection protocol that included high dose steroids, Rituximab, plasmapheresis, and intravenous immunoglobulin, but responded poorly to this regimen. A 6-month follow up biopsy continued to show lipofuscin deposition, with similar microvascular injury scores and 12-months later his creatinine remained stable but his proteinuria worsened. Patient was struggling with recurrent infectious episodes requiring hospitalizations and thus no further diagnostic or therapeutic treatments were pursued.

**Conclusions:**

Lipofuscin deposition has been reported in solid organ transplants but the significance and cause are not well understood. Several physiologic and some pathologic causes to these deposits have been reported including age, diabetes, medications and a genetic syndrome. We propose that immunologic causes such as rejection in the presence of other risk factors could potentiate the oxidative stress leading to excessive lipofuscin deposition in kidney transplants. In the case of our patient, we conclude that these deposits were likely recipient-derived, and postulate that the cumulative burden of inflammation from rejection, and underlying medical conditions led to increased lipofuscin deposition. We speculate them to be an innocent bystander.

## Background

Lipofuscin is a brown-yellow, electron-dense and autofluorescent deposit composed mainly of protein and lipids that is seen in many post-mitotic cells, and rarely in proliferating cell populations [1]. A decline in the lysosomal degradative capacity or abnormalities in lipid peroxidation leads to lipofuscin deposition (LD) in these cells [1]. This can be physiologic. For instance, LD is a characteristic manifestation of aging; thus, it is also called the “age pigment” [1–4]. However, rapid and more pronounced deposition is seen in some pathogenic processes, such as lysosomal storage diseases and neurodegenerative disorders [1, 5].

There is very limited literature in humans and in animals describing LD in the native kidneys (Table 1) [2–23]. Aging is considered to be the most common cause, and except in the case of Hermansky-Pudlak syndrome, [9–11] LD is thought to be non-pathogenic [12, 23]. Thus, LD in the kidneys of a donor is not a contraindication to kidney transplantation (KT) [4, 23]. However, there is very limited literature of LD developing after KT [8]. We present the case of a patient who underwent KT and post-transplantation, a for-cause biopsy incidentally revealed LD. The potential etiology and pathologic role of these deposits are explored.

**Table 1:** Potential etiology of lipofuscin deposits in the kidney

Causes	Commentary
*Physiological*	
Aging [6–8]	Strongest correlate of lipofuscin levels and deposition
*Congenital*	
Hermansky-Pudlak syndrome [9–11]	Diffuse tubulopathy from deposition of cytoplasmic irregular waxy brown-yellow ceroid-lipofuscin-like pigment accumulations. This is thought to be pathogenic and leads to chronic kidney disease
*Medical Conditions*	
Diabetes Mellitus [3, 8, 12]	Patients have more lipofuscin deposits that are larger in size
Hypertension [3, 12]	Lipofuscin deposits may increase in number
Uremia [13]	High oxidative stress is presumed to be the cause
Beta-Thalassemia Major [14]	This feature may be related to vitamin E deficiency secondary to fat malabsorption or hyper-consumption of Vitamin E
Vitamin E deficiency [15]	Large amount of lipid peroxides that was produced in the kidney for the period of vitamin E deficiency reacted with amino acids or protein-amino acids to produce lipofuscin by glutathione depletion.
Neurodegenerative disorders [5]	Studies have focused on increased lipofuscin deposits in neuronal cells only
*Medications and other chemicals*	
Amiodarone [2, 16, 17]	Cutaneous deposition occurs after 20 months of amiodarone use (dose: ≥160 mg/day) and is considered reversible
Aluminum Exposure [18, 19]	Chronic exposure to aluminum sulfate (33 mg/day) in rats led to lipofuscin depositions. In hemodialysis patient, increased membrane lipid peroxidation of red blood cells has been described
Analgesics [20, 21]	Seen with large doses of Acetophenetidin, Phenacetin and Acetaminophen
Estrogen [22]	Only described in rats
*Immunologic*	
Rejection	Current case

## Case presentation

### Patient information

A 48-year-old male patient with end-stage renal disease on hemodialysis underwent an expanded criteria donor KT. The donor’s age was in the early 60s and cause of death was a cerebrovascular event. The donor did not have a history of diabetes or hypertension and terminal creatinine was 1 mg/dL. Our patient received Alemtuzumab and one dose of steroids as induction therapy, and the cold and warm ischemia times were 26 h and 1 h and 15 min, respectively. His primary kidney disease was presumed to be hypertension and diabetes. Three years prior to KT, he presented with an episode of hypertensive urgency, paroxysmal atrial fibrillation and an ischemic cardiovascular accident involving the right posterior corona radiata. Following this, he was placed on anticoagulation therapy with warfarin and a few months later suffered an intraventricular haemorrhage. This left him with significant deficits, in particular, mild cognitive impairment and Broca’s aphasia. Two years after this episode, he also underwent a coronary artery bypass graft surgery. Post-operatively, he was on amiodarone for 11 months and this was stopped after KT.

### Timeline

Post-KT, our patient experienced delayed graft function and required dialysis once, within 24 h of transplantation due to hyperkalemia. Following that, he had renal function recovery and achieved a baseline creatinine of 1.2 mg/dL, with no proteinuria. He had a human leukocyte antigen mismatch of six on the A, B and DR loci and had a pre-transplantation panel reactive antibody of 0. Thus, he was maintained on dual immunosuppression: tacrolimus with a trough target of 4–8 ng/mL and mycophenolic acid 720 mg twice a day. On a routine urine test 19-months post-transplant, he was noted to have proteinuria of 0.088 g/mmol (normal ≤0.017 g/mmol); thus a graft biopsy was pursued.

### Pathology presentation

**Fig. 1:**
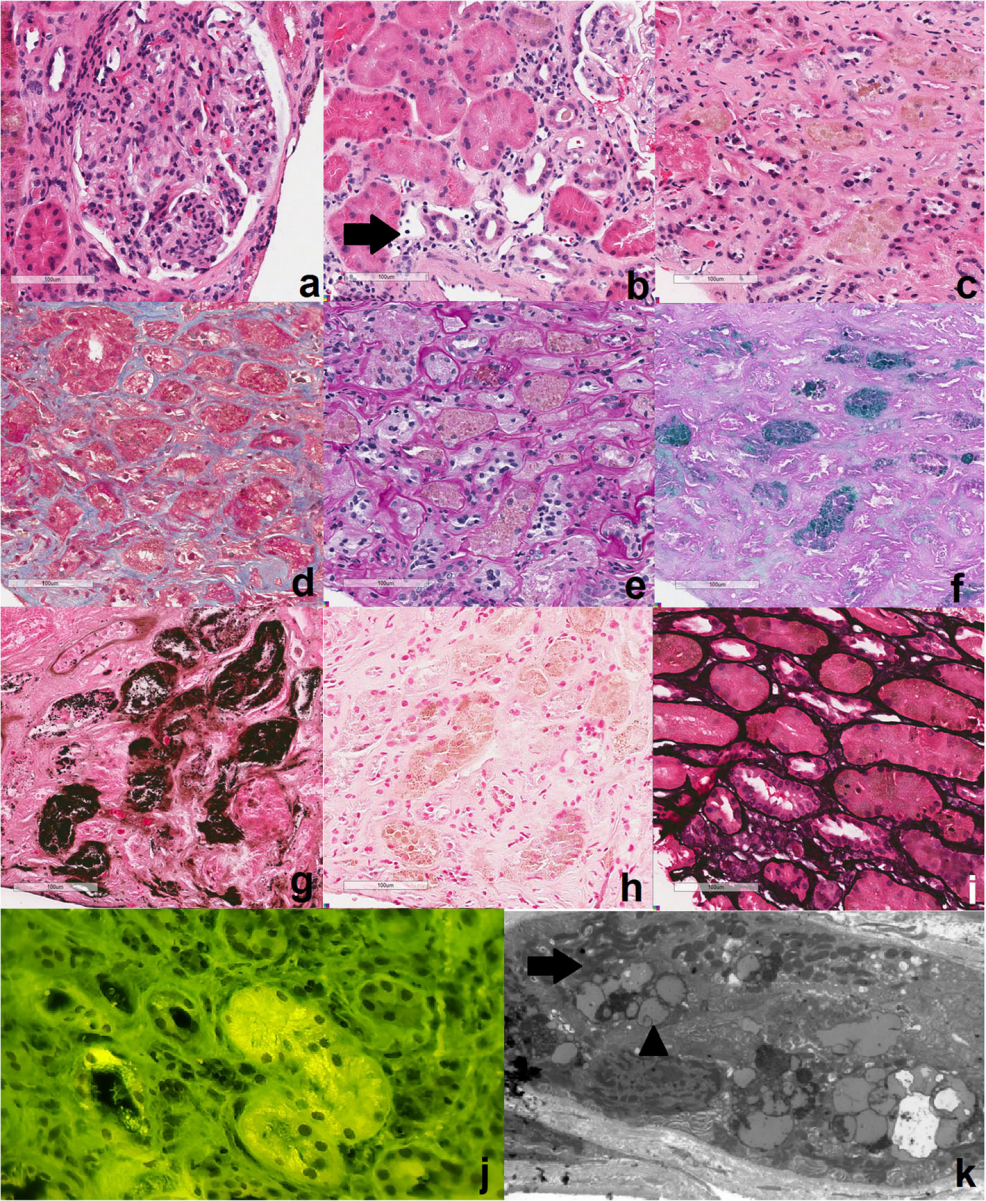
Pathology specimen of the graft biopsy of a patient who received a kidney transplant 19 month prior and now had proteinuria (200X magnification unless otherwise specified). **a** Hematoxylin and eosin: Endocapillary proliferation is seen in the glomeruli. **b** Hematoxylin and eosin: A peritubular capillary involved by numerous lymphocytes and arrow showing ptc2. **c** Hematoxylin and eosin: brown granular pigments of 2 μm on average are seen. **d** Masson-Trichrome: The granules are darker than the surrounding cytoplasm in the tubular cells. **e** Periodic-acid Schiff: Tubular epithelium with light brown to magenta granules. **f** Schmorl reaction: The blue coloration of the granules is highlighted. **g** Fontana: The black coloration of the granules is highlighted. **h** Prussian blue: the granules are negative for iron staining. **i** Jone’s silver: the granules are negative for silver staining. **j** Immunofluorescence (400x magnification): The granules show autofluorescence, exhibited by molecules with fluorophore-like property upon excitation. **k** Electron microscopy (700x magnification): Tubular cell with intracytoplasmic inclusions, compatible with lipofuscin. They have a lamellar arrangement and a granular matrix (arrow), usually surrounded by mitochondria (arrowhead)

**Fig. 2:**
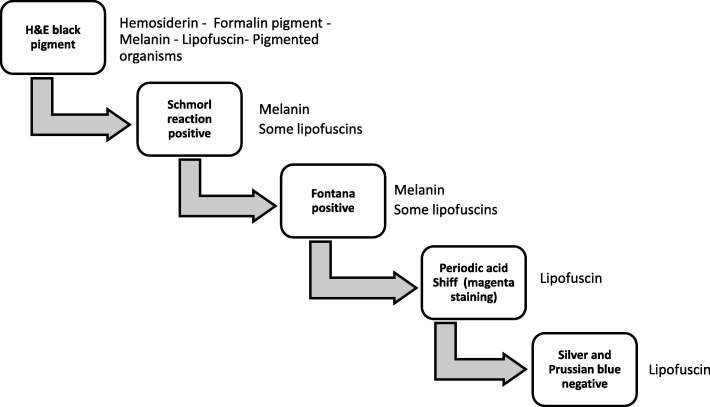
Diagnostic approach to black pigments on hematoxylin and eosin stain

The graft specimen consisted of five cores, with ¾ cortex and ¼ medulla, for a total of 76 glomeruli (three globally sclerosed) and 11 arteries. The technician noted a brown discoloration while processing the sample. Given the patient’s post-transplant status, the Banff criteria were applied [24]. The biopsy showed endocapillary inflammatory cells in the glomeruli and in the peritubular spaces, which was given a score of moderate glomerulitis (Fig. 1a) and peritubular capillaritis (Fig. 1b), but there was no significant staining for C4d in the peritubular capillaries. Also noted, were tubular changes of acute tubular necrosis or acute drug toxicity. The immunofluorescence was non-contributory. A diagnosis suspicious for acute antibody-mediated rejection was rendered. However, in addition to this, diffuse brown granular pigments in the tubular epithelial cells were seen on hematoxylin and eosin stain. The pigment was found in the cytoplasm of the tubular epithelial cells, ranging from 1 to 4 μm (Fig. 1c). Upon subsequent histologic examination with special stains, the granules were magenta on Masson Trichrome (Fig. 1d) and periodic acid Schiff (Fig. 1e), blue on Schmorl reaction (Fig. 1f), and dark black on Fontana stain (Fig. 1g). They were not reactive with iron staining by Prussian blue (Fig. 1h) and negative on Jone’s silver (Fig. 1i). The tubular cells showed autofluorescence (Fig. 1j). Electron microscopy examination revealed intracellular lamellar inclusions that had a granular matrix and were surrounded by mitochondria (Fig. 1k). All of these features were characteristic of LD (Fig. 2) [7, 25–27].

### Diagnostic assessment and therapeutic intervention

Since the transplant and during the biopsy intervals, the patient had fairly good metabolic parameters: hemoglobin A1c < 7.2%, total cholesterol < 3 mmol/L, and BMI < 25 kg/m^2^. Overall, his BP was well controlled on 2 to 3 agents. No donor-specific antibody to the human leukocyte antigen was noted except that the patient had an indeterminate antibody level against the major-histocompatibility-complex class I-related chain A (MICA). He was treated with our antibody-mediated rejection protocol which entailed three doses of steroids intravenously, two doses of Rituximab 375 mg/m^2^, and six treatments with plasmapheresis. Following this, we increased his maintenance immunosuppression regimen by increasing the target tacrolimus trough to 8–10 ng/mL and adding prednisone 5 mg to his maintenance immunosuppression. We also started monthly intravenous immunoglobulin. Unfortunately, a protocol biopsy at 6-months did not show much improvement in his inflammatory scores; although the MICA antibody was no longer present (Table 2). In conjunction with this, significant LD was still noted despite augmented immunosuppression.

**Table 2:** Comparing the pathology reports of the index for-cause biopsy done 19-months post kidney transplant and a follow-up 6-month biopsy

Index biopsy	6-month follow up biopsy
Gross description:5 cores, ¾ renal cortex, 76 glomeruli, 3 globally sclerosed, 11 arteries	Gross description: 3 cores, all cortex, 22 glomeruli, 3 globally sclerosed, 9 arteries
Banff lesion scores:^a^ • i1, t0, v0, ti1, i-IFTA1 • g2, ptc2, v0, C4d0, cg0, mm1 • ci1, ct1, ah2, cv3	Banff lesion scores:^a^ • i0–1, t0–1, v0, ti1, i-IFTA?, t0 • g2, ptc1–2, v0, C4d0, cg0–1, mm1 • ci1–2, ct1–2, ah1, cv1
Miscellaneous: lipofuscin deposition, polyoma virus immunostaining negative, immunofluorescence negative to non-specific Indeterminate antibody level against the major-histocompatibility-complex class I-related chain A	Miscellaneous: lipofuscin deposition, immunofluorescence negative to non-specific No donor specific antibody

^a^Classification based on Haas et al. Am J Transplant. 2018;18 (2):293–307

### Follow-up and outcomes

After his second biopsy, we discussed initiating the same protocol again, or using Bortezomib and/or Tocilizumab. However, the patient was tolerating augmented immunosuppression poorly, with recurrent infectious episodes requiring hospitalizations. Hence, it was decided that we will be pragmatic with his care and treat him with anti-proteinuric therapy only. At the 12-month mark post the first biopsy, his creatinine was stable and at his baseline but he had nephrotic range proteinuria of 0.4–0.5 g/mmol.

## Discussion

Renal deposits of lipofuscin in the native kidneys of humans and animals have been described in a very limited number of cases in the literature (Table 1) [2–7, 9–23]. These deposits are considered to be non-pathogenic; thus, successful transplants from donors with LD have been reported [4, 23]. These deposits have also been described in other solid organ transplants [6, 28]. In heart transplants, LD was noted in almost 50% of the patients by three years [6]. Higher serum levels of lipofuscin were tested in KT recipients [13]. In a case series of 201 living donor kidney transplant, Kawaguchi and colleagues reviewed 260 allograft biopsies and reported prevalence of LD was 58.8% [8]. However, it is not clear from the text how the authors ensured that the granules they saw were in fact lipofuscin. In addition, this cohort included living donor KT recipients in Japan that has a high rate of performing ABO incompatible transplants [29]. The implications of previous rejection episodes, desensitization protocols and heme deposits were not accounted for. Lastly, the authors do not report whether what they reported as massive LD led to gross discoloration of core biopsy samples, which was the case in our patient. At our center, while we have not stained every biopsy sample for LD, a gross discoloration of the biopsy sample necessitating investigation has not happened before.

The significance and cause of LD in a transplanted organ is not well understood. In heart transplant recipients, the presence of lipofuscin in a 12-month endomyocardial biopsy specimen was predictive of the development of angiographically confirmed cardiac allograft vasculopathy [6]. LD is thought to decrease cellular functional capacity leading to cell death by apoptosis or autophagy [30]. On the other hand, some speculate that in the presence of stress, cells are able to respond appropriately to cellular damage and initiate a protective autophagocytic response leading to LD [31]. What is peculiar about our case is the presence of an antibody to the MICA antigen that is associated with decreased renal-allograft survival [32]. MICA antigen expression is known to increase with stress, and its role in autophagy is speculated [33, 34]. However, we reviewed available biopsy samples of other patients who had a MICA antibody and did not note any LD. Thus, having an antibody to MICA alone likely does not lead to excessive LD. We, however, propose that there might be immunologic causes to LD as rejection is known to augment the oxidative stress in a transplanted graft [35]. Thus, immunologic factors may be at play and this needs to be further explored.

Kawaguchi and colleagues concluded that LD in the renal allograft tubular epithelium is not a surrogate marker for kidney allograft aging [8]. Although, older age of the recipients but not the donor was speculated to be involved in lipofuscin deposition. Despite his younger age, we postulate that the extensive LD in our patient was the result of the cumulative burden of medical co-morbidities and rejection that caused increased oxidative and inflammatory stress [6, 12, 35]. These medical comorbidities include hypertension, diabetes and a history of neurologic disease. The role of amiodarone is possible as well. However, cutaneous pigmentation typically occurs after many months of continuous treatment with amiodarone, and is reversible [2, 16]. In our patient, the history of amiodarone use was only 11-months, and it was stopped at the time of KT. We did not note any cutaneous discoloration in our patient. In addition, some have characterized amiodarone-associated inclusions to be quite different, morphologically, from lipofuscin [17]. There is low suspicion of these deposits being donor-derived as intra-operative surgical reports do not mention altered pigmentation of the transplanted kidney. The recipient who received the contralateral donor kidney is doing very well and currently does not meet the criteria of a for-indication biopsy. In addition, if donor-derived, LD decreases with time, [23] which was not the case in the two biopsy specimens performed 6-months apart in our patient. The donor was a smoker, but otherwise, had no significant past medical history or risk factors as reported in Table 1 which would have contributed to excess LD. Thus, we believe that LD in the case of our patient was likely not donor derived.

In conclusion, we report the case of a kidney transplant recipient who had LD in the transplanted graft two years after KT that are likely recipient derived. We present a thorough differential of how we concluded these deposits to indeed be lipofuscin. We also summarize the current literature and list the potential causes to LD. We propose exploring immunologic causes such as rejection as potentiating the oxidative stress that could cause more lipofuscin in the presence of other risk factors. Last, we speculate that LD is more likely to be a bystander and a manifestation of other pathogenic causes that potentiate inflammation.

## Data Availability

Not applicable

## Abbreviations

KT: Kidney transplantation
LD: Lipofuscin deposition
MICA: Major-histocompatibility-complex class I-related chain A
